# A Comparative Study on the Efficacy of Ibuprofen and Celecoxib on the Intensity of Perineal Pain Following Episiotomy: A Randomized Clinical Trial

**DOI:** 10.5812/ircmj.9980

**Published:** 2013-12-05

**Authors:** Zainab Suhrabi, Hamid Taghinejad

**Affiliations:** 1 Department of Midwifery, Ilam University of Medical Sciences, Ilam, IR Iran

**Keywords:** Ibuprofen, Celecoxib, Episiotomy, Neuralgia

## Abstract

**Background:**

Pain is a worldwide problem that often originates from disease process, and diagnostic and treatment procedures such as surgical operations.

**Objectives:**

This trial was performed to compare the effectiveness of two analgesics for the management of perineal pain caused by episiotomy.

**Materials and Methods:**

A total of 170 nulliparous women who gave birth vaginally with episiotomy between March 2009 and November 2010 were randomly assigned to receive either ibuprofen or celecoxib which were given orally every 6 or 12 hours, respectively. Pain levels were measured before the intervention, and at 1, 2, 4, 8 and 12 hours after providing the first dose on a 10-cm visual analogue scale.

**Results:**

The results showed that the two groups had no significant differences regarding demographic characteristics, maternal, neonatal, and post-delivery factors, and mean premedication pain severity. Means of pain severity were different between the two groups as patients in the celecoxib group had lower means than the other group at 1,2,4,8 and 12 hours (4.01 ± 1.8 vs. 4.46 ± 1.9, 3.17 ± 1.9 vs. 3.79 ± 1.7, 2.89 ± 1.3 vs. 2.96 ± 1.5, 2.19 ± 1.8 vs. 2.55 ± 1.4, and 1.98 ± 1.1 vs. 2.45 ± 1.2, respectively) after administration of analgesics.

**Conclusions:**

Patients who received celecoxib had lower VAS in comparison with others. Although these differences were not significant, as celecoxib has longer half-life, fewer upper GI symptoms, and is better tolerated based on the previous studies, and this study is in favor of using it.

## 1. Background

Pain is a worldwide problem that often originates from disease process, diagnostic and treatment procedures such as surgical operations (1-4). The surgical operation of widening the perineum in order to prevent severe perineal tears was first reported as far back as 1741. Today, episiotomy is one of the most frequently performed operative procedures during delivery in most parts of the world. Episiotomy has been justified on the basis that it reduces the risk of third degree perineal tears. The policy of routine episiotomy was widely practiced at the turn of the twentieth century. This policy led to high rates of episiotomy in many countries as 62.5% in The United States, 80% in Argentina, and 30% in Europe (4, 5).

Episiotomy is the most common surgical incision of the perineum among obstetrical procedures. The prevalence of episiotomy is not the same in all countries. Based on some studies, Asian ethnic women have higher risks of injuries and complications in perineal region than others as they are presumed to have smaller and tighter perineum. So the routine episiotomy may reduce the risk of perineal tearing during delivery. Medio-lateral episiotomy is usually preferred rather than a midline one because of the risk of third or fourth degree tears and also short perineum in the Asian race (6-8).

Despite the fact that episiotomy is a routine method in hospitals of Iran, there are no concise and comprehensive statistics about its prevalence. It seems that the prevalence of episiotomy is much higher than that supported by scientific evidences, and the rate of medio-lateral episiotomy is higher than median episiotomy. So, it seems that the prevalence of its complications may also be higher in Iranian women. One recent study revealed that episiotomy was performed in 97.3% of 510 primiparous women who had vaginal delivery in Tehran (8). Although this method of treatment has many benefits, it has also some complications such as dyspareunia, infection, and pain as many of women reported its pain more severe than of labor itself (9).

Episiotomy is associated with significant pain during postpartum period. Perineal pain on the first day following vaginal delivery occurs in 97% of women with episiotomies (10). Although the use of episiotomy is often debated, it remains the most common surgical procedure experienced by women (6). Pain from episiotomy is poorly treated, though it may be severe that can result in significant discomfort, interfere with basic daily activities, and adversely impact motherhood experiences (2, 3, 6). Furthermore, episiotomy may increase the risk of chronic perineal pain which is estimated to occur in 13% to 23% of women after episiotomy (2). The feeling of pain in episiotomy region may result in maternal feeling in postpartum period and affective disorders (11).Considering the adverse physiological and psychological outcomes of this complex phenomenon, effective treatment of it has significant importance regarding patient and economic perspectives. The most common method for treatment of post-episiotomy pain is the usage of systemic analgesics including non-steroidal anti-inflammatory drugs (NSAIDs), oral or intravenous opioids, as well as epidural opioids and local anesthetics (2). Meta-analysis of randomized trials of episiotomy revealed that 71.5% of women assigned to a restrictive policy would sustain posterior perineal trauma in comparison with 81.6% of whom assigned to a liberal policy indicating that posterior perineal trauma during vaginal birth is common and unavoidable (12). Suturing of perineal injury improves healing but does not alleviate the pain. Continuous subcuticular suturing has been associated with less short-term perineal pain when compared to interrupted sutures. A similar reduction in short-term perineal pain has been reported with the use of synthetic suture material as opposed to chromic catgut when performing perineal repairs (12). Codeine by-products with or without acetaminophen are effective but their complications including constipation, nausea and vomiting, abdominal pain, and dizziness limit their usage (6). NSAIDs are also useful but their gastrointestinal complications are considerable (5). Ibuprofen is one of them that prevents pain by prohibition of prostaglandins production (6) Celecoxib, a more selective cyclooxygenase 2 inhibitor (COX-2 inhibitor), is a newer analgesic that is also effective for postoperative pains (12), There are limited data in the literature regarding the usage of regional analgesia for relieving post-episiotomy pain. Pudendal nerve block (PNB) provides analgesia of the perineum. It has been used by obstetricians as an alternative to epidural analgesia during the second stage of labor (2). There are some studies about ibuprofen administration for decreasing post-episiotomy and perineal lacerations, but no study has ever compared the effect of celecoxib and ibuprofen on post-episiotomy pain. So we decided to investigate and compare this subject on nulliparous women whom were referred to Shahid Mostafa Khomeini Hospital of Ilam, Iran.

## 2. Objectives

This trial was performed to compare the effectiveness of the two above mentioned analgesics for the management of perineal pain caused by episiotomy.

## 3. Materials and Methods

This study is a single-blind clinical trial for which the researchers, after receiving a written verification with the issue No:22/4051/95/11 Oct 2008 by Ilam(western Iran) Medical University Ethics Committee, as well as a written permission signed by Head of Research Deputy of Ilam University of Medical Sciences, referred to Shahid Mostafa Khomeini (PBUH) Hospital of Ilam and, besides observing Helsinki & Belmont statements, studied 170 nulliparous women who had undergone episiotomy for delivery between March 2009 and November 2010. It should be noted that we calculated the sample size based on the following formula: 

Б = 1.5, α = 0.05, β = 0.10, power = 0.90 and µ2-µ1 = 0.75.

n = (z_ɑ_ - z_β_)^2^ * 2ɓ^2^ / (μ_2_ – μ_1_)^2^ = (1.96 – (-1.28)^2^ 2 * 1.5)^2^ / 0.75^2^ = 85

Assuming 5% dropout rate, a minimum of 178 women were needed to be recruited. Considering the sample size, and inclusion and exclusion criteria 178 eligible women were included. Eight of them declined from participation before randomization, but there was no dropout after randomization and the data of 170 women were available for analysis. The studied cases signed agreements for the trial. The inclusion criteria were: 18-35 age group, vaginal delivery with medio-lateral episiotomy, absorbable and continues sutures, single-delivery, and alive fetus. We excluded women who had known allergy to NSAIDs, history of alimentary canal disorders, underlying illness, instrumental delivery, perineal rapture (3 or 4 degree), postpartum hemorrhage, and pre-eclampsia as well as eclampsia. The cases were randomly divided into two groups of 85 women and received celecoxib (100mg) or ibuprofen (400mg). Both the medications were manufactured by Tehran Chemie Pharmaceutical Company in Iran.

 Randomization was performed by opening a sealed opaque envelope indicating the treatment allocation of A or B. Randomization sequence was created in blocks of ten and the randomized allocated treatments were placed in numbered sealed opaque envelopes by an author (HT). A numbered envelope was assigned to each studied woman according to the sequence of recruitment. The envelopes were kept in a box on the delivery ward. Celecoxib capsules were given orally at 12-hour intervals and ibuprofen every 6-2 hr. In case of feeling pain, the medication was started immediately after the stitches were finished. Then, the pain level was measured before the intervention and at 1, 2, 3, 4, 8 and 12 hours after stitching using a visual analogue scale (VAS). The person who evaluated the pain was blind to the treatment. Checklists and questionnaires were used as the investigation means. The questionnaires contained demographic specifications of the studied cases, while the checklists included the data extracted from the pain level records.

Considering the investigation goals, the content validity was used to determine the data form value. To do so, the researchers designed the data registration form for which various books, reliable scientific resources as well as a number of journals were referred. Then the questionnaire was confirmed after consultation with 5 faculty members of Ilam University of Medical Sciences. Regarding VAS, the literature review indicated that it is considered as a standard measurement tool in pain research and clinical practice, as well as a highly reliable and valid instrument for measurement of acute pain. The observed data were analyzed using version 16 of SPSS Software as well as t-test, chi-square, variance analysis, and Fisher’s Exact Test. It is notable that the data was also tested by Kolomogorov-Smirnov test which revealed normal distribution of the data.

## 4. Results

Based on the obtained findings through chi-square test , there were no significant statistical differences between the two groups of celecoxib and ibuprofen regarding demographic specifications (maternal age, body mass index), obstetric factors (gestational age, active phase duration (minute), second delivery duration (minute), third delivery duration (minute)), neonatal specifications (head circumference (centimeter)), newborn’s weight (gram), the first and fifth minute Apgar scores, and post-delivery factors (episiotomy) (recovery duration (minute), number of stitches on the skin , number of consumed stitch thread packs, and beginning time of pain after episiotomy (minute)). According to Tables 1 and 2 that demonstrate a comparison of average pain levels before intervention and at different intervals at rest between the two groups, the average pain levels in celecoxib and ibuprofen receivers were respectively as follow: before intervention (5.31 ± 1.3, 4.95 ± 2.3), one hour after intervention (3.20 ± 1.3, 3.32 ± 1.9), two hours after intervention (2.76 ± 1.9, 2.89 ± 1.3), four hours after intervention (2.57 ± 1.7, 2.34 ± 1.5), and twelve hours after intervention (1.37 ± 1.09, 1.68 ± 1.1).

**Table 1.** Comparison of Demographic Specifications, Obstetric Factors, Neonatal Specifications, and Post-delivery Factors between the Two Groups

Variable	Ibuprofen	Celcoxib
**Age, mean (SD), y**	24.45 (5.13)	25.64 (5.14)
**Body mass index, mean (SD)**	23.82 (1.3)	24.55 (2.30)
**Gestational age, mean (SD), wk**	38.89 (4.1)	39.45 (3.30)
**Active phase duration, mean (SD), min**	241.71 (53.7)	260.27 (43.30)
**Second phase duration, mean (SD), min**	41.89 (5.8)	49.45 (4.1)
**Second phase duration, mean (SD), min**	4.79 (3.9)	5.68 (3.3)
**Duration of episiotomy repair, mean (SD), min**	28.19 (5.1)	25.45 (6.6)
**Number of stitches on skin, mean (SD)**	7.89 (2.1)	8.05 (1.30)
**Number of consumed stitch thread packs, mean (SD)**	2 (0)	2 (0)
**Onset time of pain following episiotomy, mean (SD), min**	109.71 (18.7)	98.27 (20.30)
**Newborn’s head circumstance, mean (SD), cm**	33.69 (1.1)	34.18 (1.6)
**Newborn’s weight, mean (SD), g**	3456.71 (330.7)	3250.29 (213.3)
**Apgar score at 1st min, mean (SD)**	8.89 (1.1)	9.05 (0.30)
**Apgar score at 5th min, mean (SD)**	10 (0)	10 (0)

**Table 2.** Comparison of Mean Pain Intensity at Specified Time Intervals between the Two Groups.

Time, mean (SD)	Ibuprofen	Celcoxib	T	P value
**Before intervention**	4.95 (2.3)	5.31 (1.3)	-1.06	0.29
**After 1 h**	3.32 (1.90)	3.20 (1.30)	0.24	0.81
**After 2 h**	2.89 (1.30)	2.76 (1.90)	0.28	0.78
**After 4 h**	2.70 (1.40)	2.57 (1.40)	0.35	0.73
**After 8 h**	2.34 (1.50)	2.07 (1.70)	1.22	0.22
**After 12 h**	1.68 (1.1)	1.37 (1.09)	1.74	0.08

Based on t-test statistical analysis, no statistically significant differences were seen between the mean pain levels of the two groups, at the following time intervals: before intervention (P = 0.29), after one hour (P = 0.81), after two hours (P = 0.78), after four hours (P = 0.73), after eight hours (P = 0.22), and twelve hours after intervention (P = 0.08). According to Table 3, nine women of the celecoxib group and 13 ones of the ibuprofen group needed additional sedatives, but no statistically significant difference was seen between the two groups (P > 0.05). As shown in Table 4, gastric irritation and pain was the only side-effect observed in two women of the celecoxib group, while 18 patients of the ibuprofen group had the similar complication. This difference was statistically significant (P < 0.05). 

**Table 3.** Comparison of Needs to Additional Analgesics between the Two Groups

	Ibuprofen		Celcoxib	
	Percent	Frequency	Percent	Frequency
**Additional analgesic**	15.29	13	10.58	9

**Table 4.** Comparison of Side Effects between the Two Groups

Side effects	Ibuprofen		Celcoxib	
	Percentage	Frequency	Percentage	Frequency
**Heartburn**	16.36	9	2.35	2
**Nausea**	5.88	5	0	0
**Vomiting**	3.52	3	0	0
**Epigastric pain**	0	0	0	0
**Diarrhea**	1.17	1	0	0
**Constipation**	0	0	0	0
**Headache**	0	0	0	0
**Skin symptoms**	0	0	0	0
**Total**	21.17	18	2.35	2

## 5. Discussion

Intervention at different intervals of rest by oral consumption of celecoxib or ibuprofen decreased the pain caused by episiotomy. Considering the results, celecoxib was more effective than ibuprofen, but the difference was not statistically significant (P > 0.05). One study in 2008 has also demonstrated that celecoxib is more effective on the perineal pain following episiotomy, both on the move and at rest (12). It also showed that pain levels were lower following administration of celecoxib rather than diclofenac at rest, but the differences were not statistically significant on the move between them (12). Another study in 2001 revealed that ibuprofen and acetaminophen/codeine are equally effective on controlling the perineal pain following episiotomy (13). An older study in 1989 had also concluded that ibuprofen is more effective in relieving the pain caused by episiotomy than acetaminophen (14). Nine women in celcoxib group needed additional sedatives, while this rate for the ibuprofen group was 13.

As demonstrated in table 4, the only complication in the celecoxib group was gastric irritation and pain in just two women, while this adverse effect was more prevalent in the ibuprofen group as 18 ones were suffered and this difference was statistically significant. One study in 2000 showed that in comparison with other NSAIDs, celecoxib had caused fewer gastrointestinal symptomatic ulcers and less other important toxic effects (15). This research was performed to make a comparison between the effectiveness of celecoxib and ibuprofen on perineal pain following episiotomy in nulliparous women referred to Shahid Mostafa Khomeini Hospital of Ilam between 2009 and 2010. Based on the results ibuprofen consumption led to a decrease in perineal pain following episiotomy at different intervals of rest after intervention. According to our findings, celecoxib was more effective in relieving the pain than ibuprofen, although the difference was not statistically significant (P > 0.05).

Regarding our results application, in health scope, our findings may help to determine the most effective medication to control the pain following episiotomy after delivery. This is considerably important because women who undergo episiotomy continually complain of pain due to sensitivity at abdominal area so that it would be difficult for them even to sit down in early days after episiotomy. However, feeling of pain at episiotomy area causes a decline in maternal emotions towards the newborn. So, it is recommended to use analgesic celcoxib capsules with a higher half-life of 12 hours and fewer gastrointestinal side effects when compared to that of ibuprofen.
